# A technological combination of lead-glaze and calcium-glaze recently found in China: Scientific comparative analysis of glazed ceramics from Shangyu, Zhejiang Province

**DOI:** 10.1371/journal.pone.0219608

**Published:** 2019-07-11

**Authors:** Yue Wang, Yihang Zhou, Zhefeng Yang, Jianfeng Cui

**Affiliations:** School of Archaeology and Museology, Peking University, Beijing, China; University at Buffalo - The State University of New York, UNITED STATES

## Abstract

To study the relationship between the glazed pottery from southern China and the lead-glazed pottery in northern China in the Han Dynasty (202BC-220AD), 34 samples unearthed from Shangyu(上虞), Zhejiang Province have been studied by LA-ICP-AES, SEM/EDS and XRD. The results showed that these samples included the typical lead-glazed pottery, the proto-porcelain and the glazed pottery using both lead and calcium as glaze fluxing agents. Previously, the lead-glazed pottery type was considered as the main northern products during the Han dynasty while the calcium-glazed pottery type or the proto-porcelain was the representative of the south of China. However, apart from the two typical types above, a new variety of glaze categorized as the calcium-lead glaze was discovered in the samples from Shangyu. This indicates that there were technology exchanges and amalgamation of lead-glaze and calcium-glaze between the south and the north during the Han Dynasty. As a result, a new type of glazed potteries with both features was created, which had a more beautiful appearance than the proto-porcelain but perhaps had some undesirable aspects. The manufacturing process of the new variety might also lay foundations for the invention of celadon.

## Introduction

There has been a long and prosperous history of glazed ceramics production in China. Proto-porcelain, the first representative, was invented in the Shang Dynasty (1600BC-1046BC), while lead-glazed pottery was popular later in the Han Dynasty. The independent productions of the high-temperature calcium glaze in the south and the low-temperature lead glaze in the north appeared no later than the Han Dynasty(202BC-220AD). This may have formed during the Warring States Period(475BC-221BC) and lasted until the Tang Dynasty(618AD-907AD) [1]. In southern China, proto-porcelain first appeared in the Shang Dynasty [2], which experienced its peak development from the end of the Spring and Autumn Period (770BC-476BC) to the middle of the Warring States Period. In Fujian, Zhejiang, and Jiangxi Provinces, the quality of the proto-porcelain in this period was similar to that of the fine celadon after the Song Dynasty(960AD-1279AD). However, soon after the Chu State(1115BC-223BC) defeated the Yue State(2032BC-222BC) in the late Warring States Period, the production of proto-porcelain declined in the area of Yue [3]. By the Qin(221BC-207BC) and Han Dynasties, the proto-porcelain in southern China was quite different from those in the earlier period. At this time, the ceramic industry was in a recovery stage after the war and depression [4]. Some scholars believe that the later proto-porcelain should be called glazed pottery because of its loose and porous ceramic body with high water absorption [3]. In northern China, the technology of low-temperature lead-glazed pottery suddenly developed in the Han Dynasty. Nonetheless, lead-glazed potteries were only used for burial and architectural objects for some reasons [5]. The varieties of lead-glazed potteries were vibrant, which were the epitome of all aspects of material life in the Han Dynasty [6]. The earliest low-temperature lead-glazed pottery discovered presently was unearthed in the tomb of Qi in Linzi of the Warring States Period in Shandong Province [1]. Lead-glazed potteries were first popular in the tombs of the early Han Dynasty in the Central Shannxi Plain and rapidly spread to the north in the late Western Han Dynasty. The popularity of lead-glazed potteries further increased across the country in the Eastern Han Dynasty [6] and products with local characteristics were manufactured locally [7].

In terms of the manufacture techniques, the proto-porcelain and lead-glazed pottery are significantly different. The fine, compact and grey-white ceramic body of the proto-porcelain was made of porcelain stones, the aggregates of quartz and sericite, while the ceramic body of a lead-glazed pottery was made of the fusible clay with more iron and titanium impurities [8]. Most of their bodies turned red after firing in an oxidizing atmosphere and the iron impurities in the ceramic body materials affect the sintering strength [9]. Furthermore, the proto-porcelain surface was glazed with a calcium glaze formula, and the glaze layer was usually thin. In the initial production stage, the thickness of the glaze layer was uneven, often resulting in thick glaze spots or wavy lines [10]. Compared with the glazed pottery, the glaze of proto-porcelain was tightly combined with the body, in which limestone or plant ashes served as fluxing agents, and iron was the main colorant. The firing temperature was around 1,200 to 1250°C [11]. Nevertheless, lead was used as the main fluxing agent in the lead-glazed pottery, which was fired at about 700–900°C. Copper and iron were used as the main colorant in the yellow or green lead-glazed potteries. Some of them use a secondary glazing technique, i.e., the potteries were glazed more than once [12].

Shangyu, located in eastern Zhejiang, is an important proto-porcelain production area in China. In the late Eastern Han Dynasty, the fine celadon was created at the Xiaoxiantan Yue Kiln in Shangyu for the first time [13]. Celadon was bound to be first successfully fired in Shangyu because of the long history of the ceramic industry, superior geographical location and economic development here [4]. To observe the development of celadon technology in Shangyu and further explore the development process from the proto-porcelain to the fine celadon, green and yellow green glazed ceramics unearthed from the tombs of the Han Dynasty in Shangyu were selected in this study for scientific analysis. Among these samples, a special kind of ceramics takes a large proportion, of which the glaze is smooth and compact.

## Materials and methods

### Sample information

**Fig 1:**
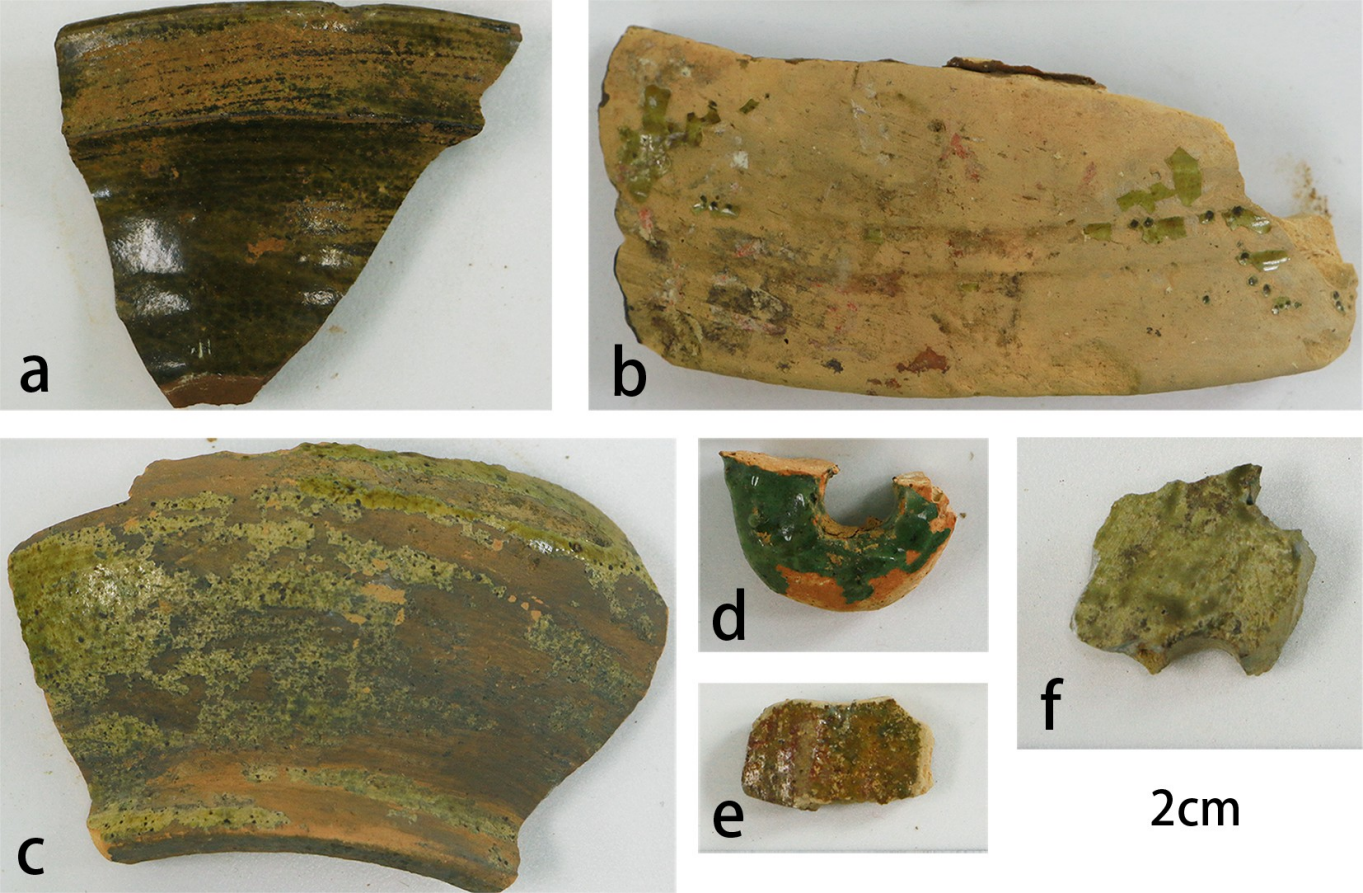
The appearance of the representative samples. (a) The sample No. 24 with dark green glaze and hard ceramic body, (b) The sample No.14 with yellow green thick glaze, (c) The sample No. 04 with yellow green thin glaze and loose ceramic body, (d)The sample No. 23 with emerald glaze, (e) The sample No. 02 with dark green glaze, (f) The sample No. 19 with yellow green glaze with plots.

There are 34 samples dated from the late Western Han Dynasty to the early and middle Eastern Han Dynasty. All samples are temporarily stored at room temperature in the laboratory of School of Archaeology and Museology, Peking University. Each sample is packed separately in a plastic bag. They are provided by Shangyu Museum, Zhejiang Province, China and have not been publicly displayed yet. Details are shown in Table 1. The appearances of some typical samples are shown in Fig 1 and other samples are shown in S1 File.

**Table 1:** Sample information.

No.	Excavation details	Ceramic body Color	Glaze Color
01	4481A4477M60: 7熏	Red, grey	Dark green
02	4449A4445 92SYHTSM11: 11 vase with four orifices	Yellow	Dark green
03	4460A4456 upon M52: 3 簋	Yellow	Dark green
04	4579A4575 76 upon M74: 1 little caldron	Red	Yellow green
05	4557A455389-65 vase with five orifices	Grey	Dark green
06	5522A5518 84 upon M250: 10鐎斗	Red, grey	Yellow green
07	5541A5537 upon M57: 3钵	Red	Dark green
08	5518A5514 upon M52: 4 amphora	Yellow	Yellow green
09	5530A5526 84 upon M239: 2 rim of the pot	Red	Yellow green
10	5550A5546 upon M52: 9 钵	Yellow	Yellow green
11	5560A5556 84 upon M249: 2 rim of the pot	Red	Yellow green
12	5558A555484 upon M129: 3 amphora	Red	Dark green
13	5824A5820 84 upon M250: 7簋	Red	Yellow green
14	5893A5889 92 upon M48: 3 drench dish	Red	Yellow green
15	6265A626193SYM31: 10 vase with five orifices	Grey	Yellow green
16	6343A6339 vase with many orifices	Red	Yellow green
17	05SMM65鐎斗	Red	Yellow green
18	6581A657790-81 rim of the vase with five orifices	Red	Dark green
19	32 2 90–464 熏	Grey	Yellow green with plots
20	M301: 1 board	Red, grey	Yellow green
21	09 upon Changlou M15: 1 board	Yellow	Yellow green
22	90–278 board	Red	Yellow green
23	D52M8: 7 glazed锺	Red	Emerald
24	15 锺	Red	Dark green
25	92SYHTSM13 rim of the pot	Yellow	Dark green
26	92SYHTSM24: 2 oven	Red, grey	Dark green
27	92SYHTSM24: 3 drench pot	Red, grey	Dark green
28	84 upon M60: 6 little pot	Red	Dark green
29	84 upon M279: 24 小碗	Grey	Yellow green
30	84 upon M250: 24 ear cup	Red	Yellow green
45	G5M1: 1 proto-porcelain pot	Grey	Yellow green
46	76 upon M70: 20 熏	Red	Yellow green
60	D45M8: 7	Red	Yellow green
61	D28M10: 11	Yellow	Yellow green

### Analytical methods

#### Laser ablation inductive coupled plasma atomic emission spectrometry (LA-ICP-AES)

The samples glazes were quantitatively analyzed for their chemical compositions using LA-ICP-AES. A LEEMAN-Prodigy ICP-AES with a NEW-WAVE laser ablation system was used to carry out the analysis. Before the test, the samples surfaces were clean with alcohol.

The operating conditions for the LA-ICP-AES system are as follows: 1) RF generator: 40.82 MHz; 2) RF Power: 1.1 kw; 3) Argon flow rate: Plasma: 20 l/min; 4) Auxiliary pressure: 0 psig; Nebuliser pressure: 30 psig; 5) Laser: Nd-YAG; 6) Laser mode: Q-switched; 7) Laser Wavelength: 266 nm; 8) Output energy: 3.5~7mJ; 9) Facular aperture:240~350 μm; 10) Helium flow rate: 1120 ml/min. The test results of the standard samples are shown in S1 Table. The detection limit of the instrument is 1-10ppm.

#### Scanning electron microscopy (SEM) with energy dispersive spectroscopy (EDS)

Scanning electron microscope (Hitachi TM3030) was used to observe the cross sections of the samples, and to analyze the chemical compositions of micro areas. A small piece of each representative sample was taken off and was solidified in epoxy resin. Then the cross section was grinded out and polished. The test time of EDS was no less than 80 seconds. The working distance is 6.5–9.5mm and the high voltage is 15kV. The detection limit of the instrument is 0.1%.

#### X-ray diffraction (XRD)

Four ceramic bodies of the samples were tested by XRD (OLYMPUS Terra). Before the test, a small amount of the ceramic body of each sample was taken off and grinded into fine powder. The test conditions are as follows: Tube style: Co. Tube voltage: 30kV. Tube current: 300uA. Vibrational frequency: 6000Hz. Angle range: 5°-55°(2θ). The step:0.04°(2θ).

## Results and discussion

### Fluxing agents

**Fig 2:**
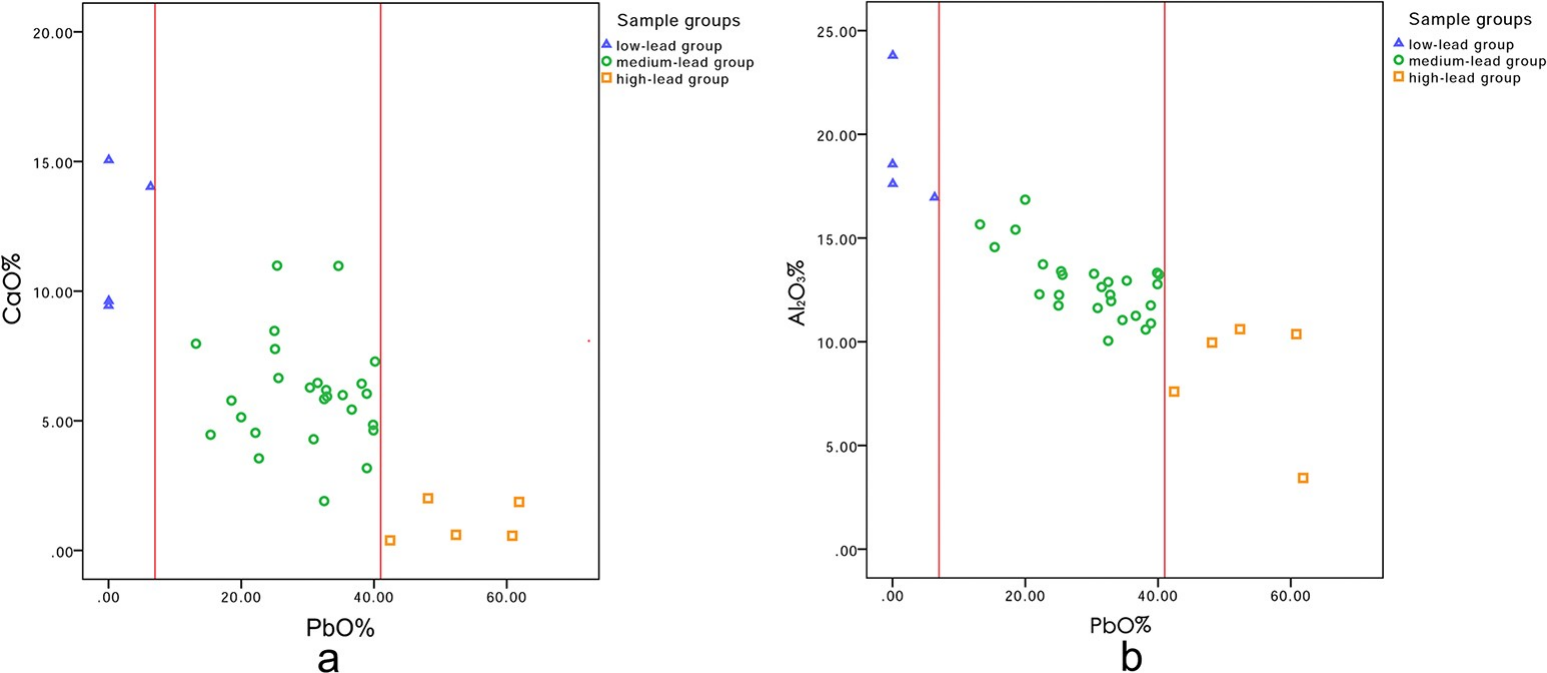
Distribution map of lead content in glaze of samples. (a) The lead-calcium distribution map. (b) The lead-aluminum distribution map.

The main fluxing agent of the glazes are lead oxide and calcium oxide, as shown in Table 2. Lead-calcium and lead-aluminum diagrams were shown in Fig 2. The lead oxide contents of the glazes can be divided into three regions, referring to three groups. The low-lead group barely contains lead oxide, including the samples No. 15, 19, 22-yellow, and 45. The lead oxide content of No. 22-yellow glaze is about 6.4%, which should be caused by the pollution of its green part. The green-glaze part of the sample No. 22 contains more lead oxide (13.2%) and the green-glaze might flow around during firing. The content of lead oxide in the high-lead group is higher than 42%, including No. 02, 14, 23, 60, and 61. The rest of the samples belong to the medium-lead group with lead oxide content ranging from 7% to 42%. From Fig 2A, it can be seen that there is a negative correlation between lead oxide and calcium oxide content, indicating that the two sources are different, i.e., adding lead oxide will reduce the addition of calcium oxide accordingly. Lead oxide was used as a fluxing agent in the high-lead group, while calcium oxide was used as a fluxing agent in the low-lead group, and both were used in the medium-lead group. The glazes of the samples in medium-lead group are categorized as the calcium-lead glaze type.

**Table 2:** The LA-ICP-AES analysis results of sample glaze (%).

No.	SiO_2_	Al_2_O_3_	Fe_2_O_3_	MgO	CaO	Na_2_O	K_2_O	P_2_O_5_	TiO_2_	MnO	CuO	SnO_2_	PbO
**1**	47.19	15.41	3.14	1.15	5.78	1.04	5.60	0.39	0.65	0.06	0.17	0.06	18.52
**2**	41.63	7.60	1.86	1.03	0.39	0.34	1.85	0.08	0.47	0.01	1.67	0.44	42.43
**3**	38.06	11.25	3.30	0.78	5.43	0.51	2.60	0.30	0.48	0.02	0.10	0.04	36.65
**4**	37.04	10.88	3.96	0.96	3.17	0.55	2.08	0.24	0.51	0.03	1.15	0.02	38.93
**5**	39.64	13.40	3.86	1.51	10.98	0.45	2.33	0.99	0.54	0.11	0.19	0.08	25.40
**6**	42.54	13.23	3.74	1.08	6.65	0.68	3.95	0.52	0.63	0.06	0.63	0.04	25.60
**7**	48.20	10.05	2.80	0.56	1.90	0.39	2.39	0.15	0.45	0.02	0.20	0.04	32.49
**8**	29.66	13.24	4.87	0.96	7.28	0.30	1.70	0.38	0.56	0.04	0.15	0.06	40.16
**9**	37.62	12.27	3.34	1.35	6.19	0.50	4.42	0.52	0.55	0.05	0.04	0.03	32.80
**10**	33.37	13.32	3.67	0.87	4.84	0.34	2.19	0.34	0.62	0.04	0.05	0.02	39.87
**11**	32.80	12.77	4.18	1.35	4.63	0.31	1.53	0.57	0.59	0.10	0.70	0.03	39.92
**12**	53.56	14.56	4.68	0.76	4.46	0.87	4.08	0.28	0.81	0.03	0.01	0.07	15.38
**13**	35.55	12.94	3.97	1.24	5.99	0.43	2.89	0.33	0.56	0.04	0.31	0.03	35.29
**14**	31.58	9.96	3.14	0.69	2.01	0.37	1.82	0.15	0.48	0.02	1.21	0.02	48.14
**15**	53.55	18.56	6.08	3.44	9.44	1.20	5.74	0.55	0.91	0.11	0.00	0.03	0.01
**16**	42.52	11.63	3.95	1.18	4.29	0.49	3.66	0.37	0.57	0.06	0.09	0.03	30.90
**17**	44.16	11.74	2.87	1.05	8.47	0.63	4.04	0.56	0.58	0.13	0.09	0.18	25.00
**18**	50.22	13.73	2.79	1.15	3.55	0.78	3.47	0.44	0.64	0.04	0.11	0.09	22.66
**19**	50.35	17.61	6.45	2.71	15.06	0.73	3.94	1.03	0.93	0.28	0.00	0.09	0.04
**20**	34.17	10.59	3.01	1.08	6.43	0.62	4.14	0.38	0.40	0.09	0.27	0.03	38.14
**21**	33.89	11.04	4.32	1.11	10.97	0.20	1.83	0.60	0.55	0.09	0.39	0.03	34.64
**22-green**	50.81	15.66	3.75	1.90	7.97	0.63	3.62	0.95	0.73	0.10	0.01	0.10	13.18
**22-yellow**	48.66	16.96	4.00	2.68	14.03	0.58	3.65	1.00	0.86	0.20	0.03	0.12	6.35
**23**	26.87	3.43	1.21	0.48	1.87	0.15	0.18	0.11	0.24	0.04	2.69	0.64	61.86
**24**	38.70	13.28	3.54	1.01	6.28	0.54	4.67	0.39	0.58	0.05	0.06	0.06	30.35
**25**	38.39	12.88	3.82	0.93	5.84	0.52	3.51	0.44	0.46	0.03	0.09	0.05	32.49
**26**	41.71	12.26	4.54	1.52	7.77	0.90	3.76	0.90	0.63	0.07	0.05	0.19	25.08
**27**	34.87	11.75	2.61	1.44	6.04	0.49	1.78	0.68	0.51	0.13	0.20	0.04	38.91
**28**	47.73	16.85	3.12	1.19	5.14	0.80	3.29	0.27	0.79	0.09	0.02	0.05	19.99
**29**	38.91	11.96	4.57	1.45	5.94	0.40	2.27	0.55	0.57	0.08	0.04	0.03	32.93
**30**	38.12	12.64	4.97	1.11	6.46	0.44	2.67	0.50	0.73	0.08	0.25	0.02	31.50
**45**	47.55	23.80	9.35	2.17	9.62	0.73	3.84	0.83	1.24	0.13	0.01	0.09	0.04
**46**	49.93	12.29	3.95	1.30	4.54	0.81	2.62	0.55	0.71	0.07	0.37	0.03	22.13
**60**	21.63	10.37	3.93	0.59	0.57	0.16	0.56	0.04	0.57	0.03	0.47	0.02	60.83
**61**	29.14	10.60	3.89	0.59	0.60	0.35	0.93	0.03	0.64	0.02	0.15	0.02	52.35

**Fig 3:**
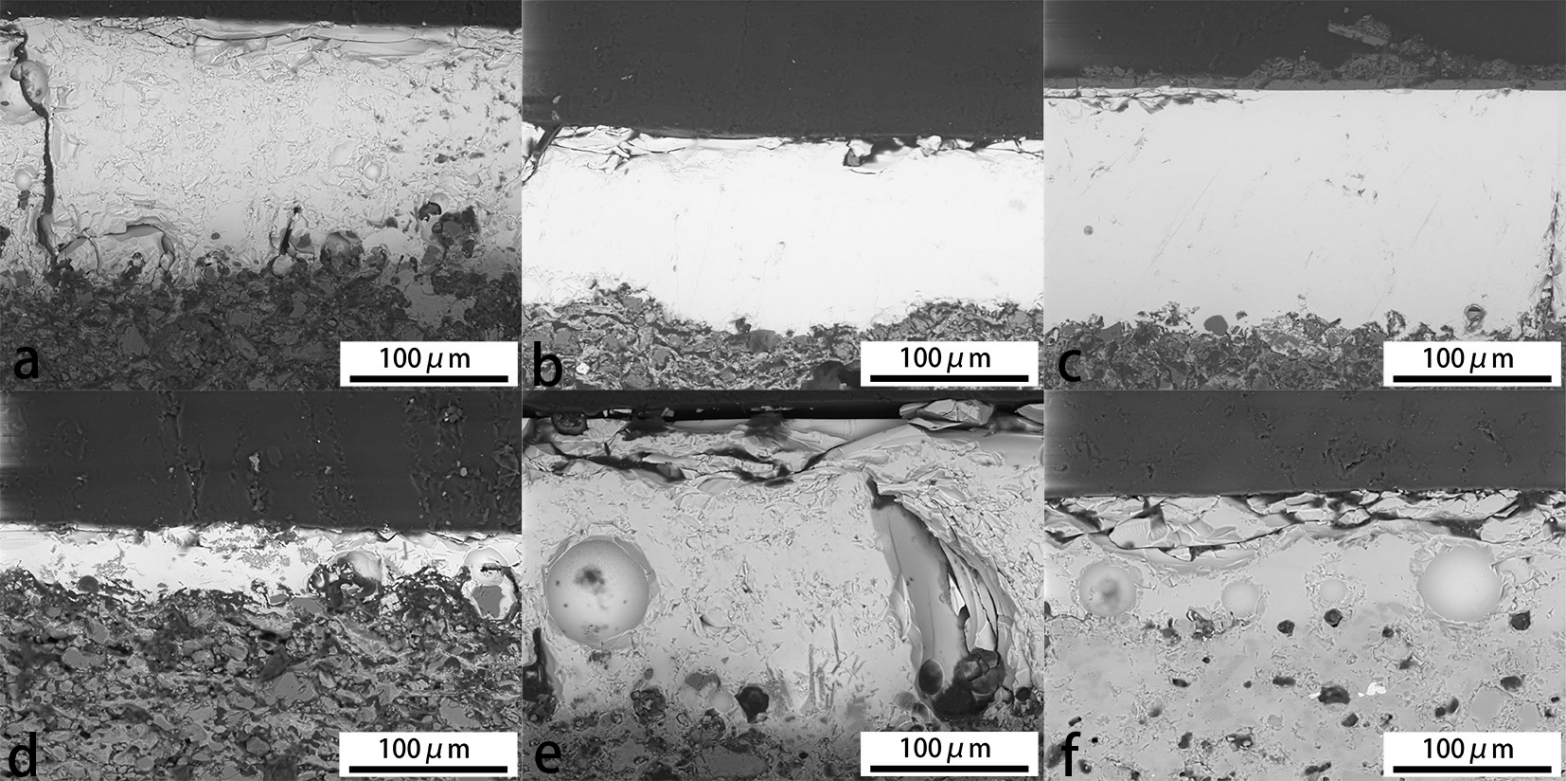
SEM images of samples glaze layers (500X). (a) The sample No. 02. (b) The sample No. 14. (c) The sample No. 23. (d) The sample No. 04. (e) The sample No.24. (f) The sample No.19.

**Fig 4:**
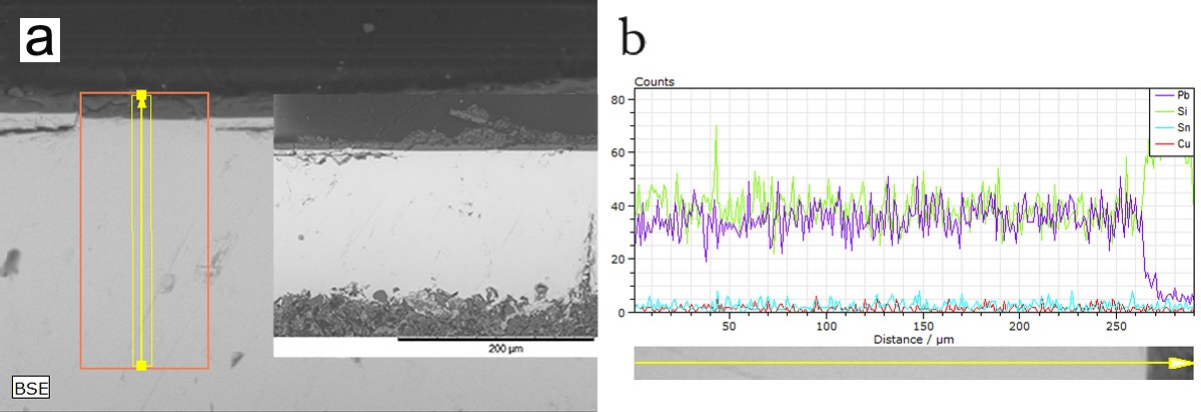
The test area and results of sample No. 23 glaze. **(**a) Linear test area of the glaze in sample. (b) Linear variation of the glaze elements in sample.

Lead oxide has a strong fluxing effect, which impacts the thickness and the quality of the glaze. Fig 3. shows that the glaze layers of the high-lead group are thicker than the other groups, ranging from 100 to 150μm. Among them, the glaze layers of the samples No. 14 and 23 are more uniform and purer. The sample No. 02 has more glaze cracks and impurities. Out of the glaze layer of No. 23, there is a thin layer with a thickness of about 6μm. Compared to the glaze layer, the silicon content is higher and the lead content is lower, shown in Fig 4A and 4B, which indicates the thin layer was probably caused by corrosion. In the medium-lead group, the glaze layer of the sample No. 04 is thinner, about 35μm, and the thickness is more uneven. The glaze layer of No. 24 is thicker, about 210μm, and there are more cracks and bubbles in both samples than the samples of high-lead group. In the low-lead group, the glaze layer of No. 19 is only 80μm with many bubbles. The amount of air bubbles in the glaze of the high-lead group is less than that of the medium-lead group, and the low-lead group has the highest amount, which is consistent with the explanation [14] that the addition of lead makes the glaze more fluid and the air easier to escape during the firing process.

### Colorants

Copper and iron are the main colorants of these glazes. The main colorant of the glazes of No. 02, 14, and 23 in the high-lead group is copper because of its high content. Since a small amount of tin was detected in the samples, it is speculated that the copper colorant was from scrap tin bronze or their rust [15]. As for the medium-lead group and the low-lead group, the copper contents in the glaze of most samples are very low. Thus, the reason why most of the samples in the medium-lead group seem yellow green is different from that of the high-lead group. The colorant is not copper but iron, like the proto-porcelain, which is also inherited by fine celadon [16]. The difference of glaze colors between yellow green and green is due to the change of iron valence [17], which is decided by the firing temperature and atmosphere [18]. This also means that, unlike the northern lead glaze, this type of calcium-lead glaze was fired in a reducing atmosphere.

### Ceramic bodies

There are differences between the ceramic bodies of the three groups. The ceramic body compositions of the 6 representative samples were analyzed by SEM/EDS, as shown in Table 3. In high-lead group, the raw material of the ceramic body have higher Al_2_O_3_ and Fe_2_O_3_ contents, which was more similar to the fusible clay. Though the specific sources of the raw materials and the workshops of the products cannot be confirmed now, a different clay source is affirmative. The use of more strongly colored glazes enabled the use of more iron-rich clay, which would not be suitable for proto-porcelain. The results of the sample No. 19 reflect its raw material was porcelain stones [4,19–20]. The compositions of samples in the medium-lead group are close to those in the low-lead group, except the higher Fe_2_O_3_ contents. Thus, it is speculated that the raw materials of the ceramic bodies in the samples from the medium-lead group were also porcelain stones, but the elutriation was insufficient. From the perspective of the appearance, high iron content resulted in darker color and lower sintering strength of the ceramic body. Therefore, the ceramics bodies of the low-lead group are grey while the other samples are in different colors.

**Table 3:** The results of ceramic bodies tested by SEM/EDS(wt.%).

No.	Sample group	Na_2_O	MgO	Al_2_O_3_	SiO_2_	K_2_O	CaO	TiO_2_	Fe_2_O_3_
**02**	High-lead	0.2	0.6	23.8	67.4	0.6	0.2	1.6	5.6
**14**	High-lead	1.0	1.2	24.5	63.4	2.8	0.5	0.9	5.5
**23**	High-lead	0.7	0.9	21.6	66.8	2.2	0.4	1.3	6.0
**04**	Medium-lead	0.1	0.2	15.4	76.8	2.6	0.7	0.4	3.6
**24**	Medium-lead	0.6	0.2	19.0	70.9	3.0	1.1	0.9	4.3
**19**	Low-lead	0.7	0.6	15.1	78.7	2.2	0.2	0.8	1.7

### Firing temperature

Lead oxide has a stronger fluxing effect than calcium oxide and can greatly reduce the firing temperature. To accommodate to the different formulas of the glazes, the firing temperatures have to be adjusted, which might reflect on the mineral phases of the bodies. The ceramic body of the samples No.04,19, 23, and 24 were tested by XRD. Quartz, rutile and mullite were detected, as shown in Table 4. The formation of mullite requires a temperature higher than 1200°C, which means the samples No.19 and 24 were fired at a high temperature. The firing temperature of the sample No. 04 and 23 are much lower since no mullite signal was detected. This result shows that the firing temperature varies greatly between the low-lead group and the high-lead group while the medium-lead group seems more complicated. The overall content of Al_2_O_3_ and SiO_2_ of the sample No. 04 (92.2wt.%) is higher than that of the sample No. 24 (89.9wt.%), while the PbO content of the glaze of the sample No. 04 (38.9wt.%) is much higher than that of the sample No. 24 (30.4wt.%). These two aspects indicate a contradictory and incompatibility between the presumed high sintering temperature of the body and the low melting temperature of the glaze, which explains the underfiring loose body of No.04 and its crystal phases similar to the high lead group.

**Table 4:** The XRD test results of the ceramics bodies (wt.%).

No.	Sample group	Quartz	Rutile	Mullite	Global amorphous stuff
**04**	Medium-lead	86.1	8.1		5.8
**19**	Low-lead	72.2		21.2	6.7
**23**	High-lead	85.5	8.9		5.6
**24**	Medium-lead	67.3		24.9	7.8

### The significance of the calcium-lead glaze

According to the results of the fluxing agents, the colorants, the ceramic bodies and the firing temperature, the samples of high-lead group are in line with the characteristics of the traditional northern lead-glazed potteries, suggesting that they came from the north, either technologically or commercially. The samples of the low-lead group belong to the high-temperature calcium glaze system of proto-porcelain in southern China. However, as their manufacture quality is not comparable to fine porcelain, they are all categorized as the proto-porcelain.

The medium-lead group is the most special group. The colorant is iron and the raw material of the ceramic bodies is porcelain stone, which are the local tradition in Shangyu. From the perspective of the firing conditions, the manufacturers might lose accurate control of the firing temperature or, in other words, sometimes failed to adjust the body formula to accommodate to the glaze firing temperature.

As for the aesthetic appearance, the quality of the ceramics bodies varies, while the calcium-lead glaze is significantly better than that of the proto-porcelain. The glaze layer is clear and flat with bright color. It is closer to the appearance of fine celadon later invented in Chinese ceramic history. However, due to its relatively high lead oxide content, it is still a kind of low-temperature glaze. The lead content in the glaze layer is significantly lower than that of the traditional northern lead glaze. Compared to the high-lead glazes analyzed, the lead oxide content is about half lower. Additionally, the calcium oxide content of the glaze is significantly higher, which was probably influenced by the high-temperature glaze formula. Therefore, this kind of glaze is supposed to be a technological combination of lead-glaze in northern China and calcium-glaze in southern China.

Moreover, the production of these special glazed potteries can be viewed as an exploration for more aesthetic appearance of the glaze at the time right before the born of celadon. This special variety is the only calcium-lead-glaze product found so far. It shows that in the process of developing the proto-porcelain to fine celadon, potters in Shangyu had made an alternative attempt. The calcium-lead glaze was an example that the potters in Shangyu learned different technology from other places and their cognition degree of the role of different components in glaze gradually increased. To improve the appearance quality of the proto-porcelain, the potters integrated the northern and southern glaze technology and invented this new glaze variety with a more beautiful appearance. However, although the appearance of this kind of calcium-lead glaze is significantly better than that of the proto-porcelain during the same period, the incompatibility between the relatively low firing temperature of the glaze and high firing temperature of the body made by local porcelain stones might be a driving factor for potters to turn their focus back on high-temperature glazed ceramics with similar appearance as the calcium-lead glaze. The tentative experience of manufacturing this kind of potteries might also reveal other disadvantage of the PbO addition technique and lead to the search for a high temperature glaze formula compatible with the body made by local porcelain stones. Therefore, to some extent, the production of these special glazed potteries laid a foundation for the manufacture of fine celadon in the late Eastern Han Dynasty.

Furthermore, this kind of glazed pottery is not simply an experimental variety because there is a large amount of potteries of this kind that have been found in the tombs of Han dynasty in Shangyu. From the late western Han dynasty to the early and middle eastern Han dynasty, the lead pottery spread across the country while proto-porcelain was still manufactured in south of China. The calcium-lead glaze ceramics may be also considered as the localization of lead-glazed pottery in Shangyu. The variety seems to be very popular and served as the mainstream products for burial practices during this transitional time before the celadon was invented.

## Conclusion

Most of the 34 samples tested in this paper are glazed potteries from Shangyu in the Han Dynasty. The samples can be divided into three groups according to the lead content of glaze. Five of them are traditional lead-glaze potteries, four are proto-porcelains and the rest are calcium-lead glazed potteries.

In the high-lead group, the lead content in the glaze is more than 42%. The main colorant of most samples is copper or iron and the firing temperature is low. The ceramic body is red or yellow and loose with impurities and holes, making from a kind of material similar to fusible clays yet different from that of the other two groups. All these features suggest that these samples are similar to the northern lead-glazed pottery. Combined with the analysis of lead-glazed potteries by relative research, it can be concluded that these high-lead glazed potteries were probably introduced from the north to Shangyu, reflecting the unification of the Han Dynasty and frequent communication between the north and the south.

The low-lead group is proto-porcelain, of which the glaze contains little lead. Calcium oxide was used as the principal fluxing agent and iron was used as the colorant. The grey-white ceramic bodies of the low-lead group were made from porcelain stones, which was a typical feature of local proto-porcelain. The firing condition was a reducing atmosphere under a high temperature.

The medium-lead group accounts for the majority of the samples. Their ceramic body materials are porcelain stones, and the main colorant of the glaze is iron. The lead content of the glaze is significantly lower than that of the lead-glaze pottery, while the calcium and potassium contents are lower than the proto-porcelain. This new variety of calcium-lead glaze was probably based on the traditional manufacture of the proto-porcelain in the south, while the local potters learned the technique of using lead as a fluxing agent from the north to improve the products appearance. This kind of calcium-lead-glaze products overcame the disadvantages of both the uneven thickness of the glaze layer of proto-porcelain and the large area of unglazed ceramic bodies. The new glaze technique can produce glazed objects with smooth, clear, and full glazes. Thus, these products became very popular locally at that time.

In short, this group of products is a new variety developed by the potters in Shangyu based on the proto-porcelain’s traditional manufacture techniques while they learned to add lead oxide to the glaze from northern China for pursuing better appearances. However, for some undesirable aspects of this technique, one of them probably being the incompatible firing temperatures of the glaze and the body, this technique was abandoned. Not for long after the prevalence of the calcium-lead glazed potteries in Shangyu, a new similar product with high temperature glaze, known as celadon, was invented in the late Eastern Han dynasty and took place of the calcium-lead glazed ones.

## Supporting information

S1 Fig The XRD test spectra of the ceramic bodies. (TIF)

S1 File The photos of the samples. (ZIP)

S1 Table The LA-ICP-AES test results of the standard samples. (DOCX)
