# Supplementary figures and images for: A technological combination of lead-glaze and calcium-glaze recently found in China: Scientific comparative analysis of glazed ceramics from Shangyu, Zhejiang Province
Source: PLoS One. 2019 Jul 11;14(7):e0219608. doi: 10.1371/journal.pone.0219608 (PMC6622541; doi:10.1371/journal.pone.0219608)

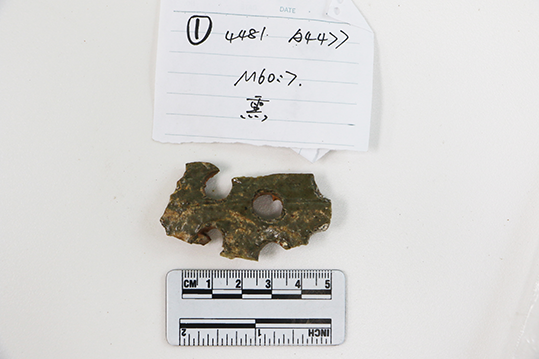

Supplement: S1 File — (ZIP) [file pone.0219608.s002.zip › S1 Fig/No.01.tif]

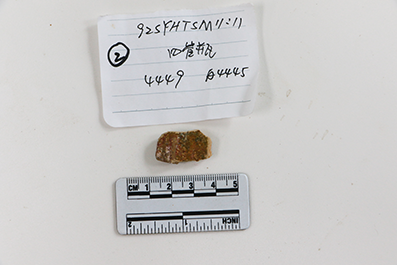

Supplement: S1 File — (ZIP) [file pone.0219608.s002.zip › S1 Fig/No.02.tif]

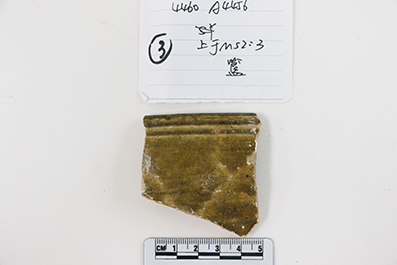

Supplement: S1 File — (ZIP) [file pone.0219608.s002.zip › S1 Fig/No.03.tif]

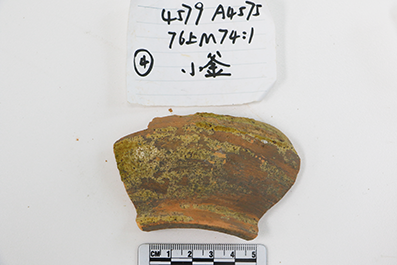

Supplement: S1 File — (ZIP) [file pone.0219608.s002.zip › S1 Fig/No.04.tif]

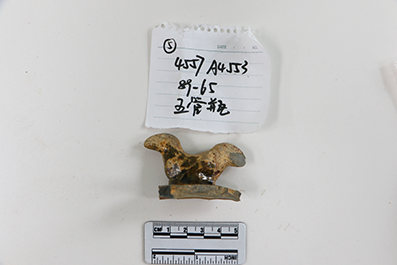

Supplement: S1 File — (ZIP) [file pone.0219608.s002.zip › S1 Fig/No.05.tif]

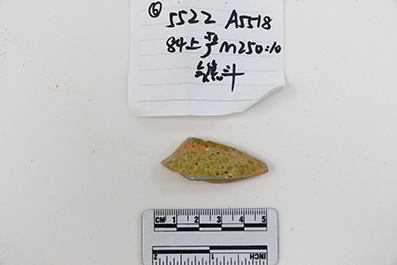

Supplement: S1 File — (ZIP) [file pone.0219608.s002.zip › S1 Fig/No.06.tif]

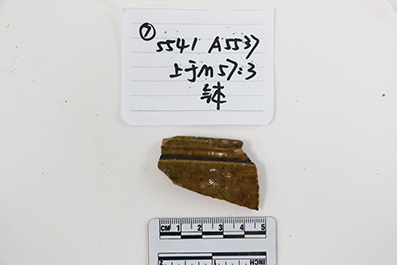

Supplement: S1 File — (ZIP) [file pone.0219608.s002.zip › S1 Fig/No.07.tif]

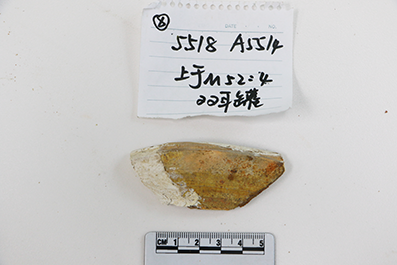

Supplement: S1 File — (ZIP) [file pone.0219608.s002.zip › S1 Fig/No.08.tif]

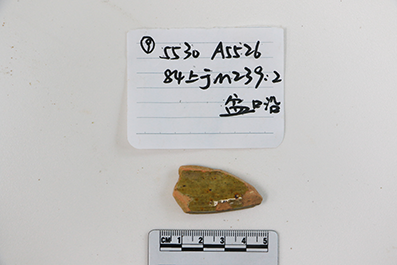

Supplement: S1 File — (ZIP) [file pone.0219608.s002.zip › S1 Fig/No.09.tif]

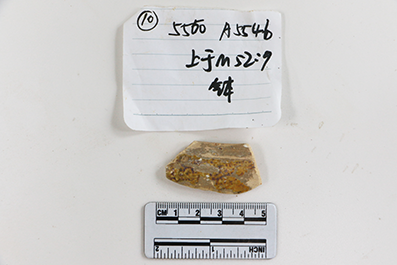

Supplement: S1 File — (ZIP) [file pone.0219608.s002.zip › S1 Fig/No.10.tif]

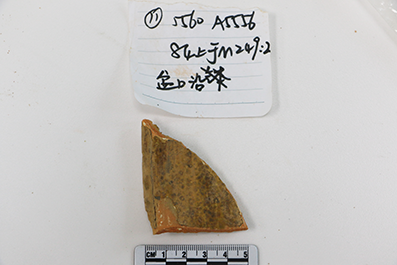

Supplement: S1 File — (ZIP) [file pone.0219608.s002.zip › S1 Fig/No.11.tif]

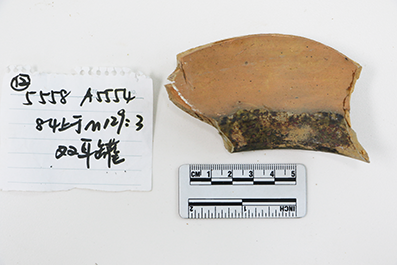

Supplement: S1 File — (ZIP) [file pone.0219608.s002.zip › S1 Fig/No.12.tif]

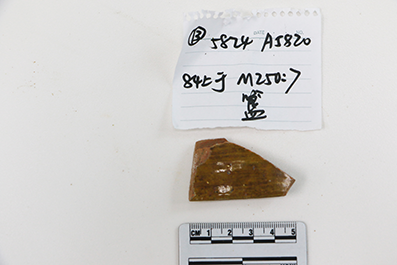

Supplement: S1 File — (ZIP) [file pone.0219608.s002.zip › S1 Fig/No.13.tif]

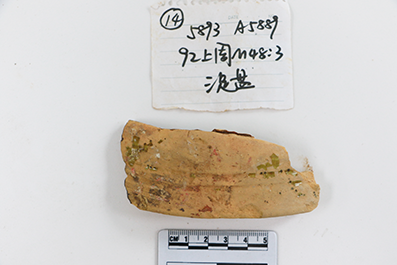

Supplement: S1 File — (ZIP) [file pone.0219608.s002.zip › S1 Fig/No.14.tif]

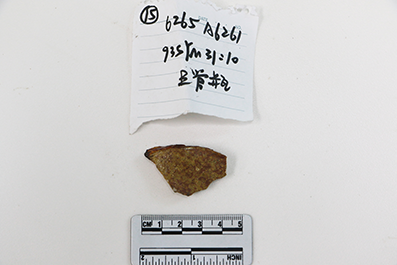

Supplement: S1 File — (ZIP) [file pone.0219608.s002.zip › S1 Fig/No.15.tif]

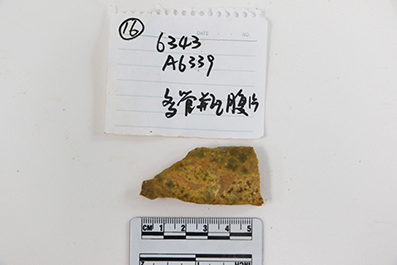

Supplement: S1 File — (ZIP) [file pone.0219608.s002.zip › S1 Fig/No.16.tif]

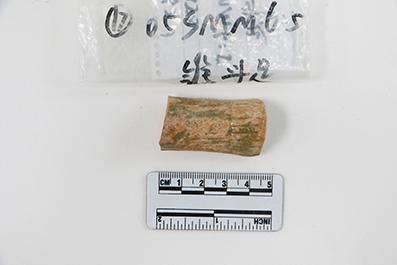

Supplement: S1 File — (ZIP) [file pone.0219608.s002.zip › S1 Fig/No.17.tif]

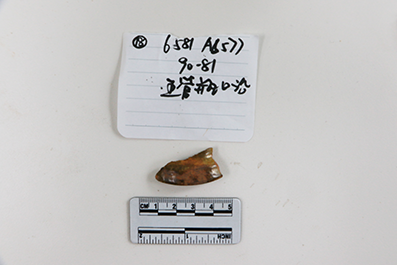

Supplement: S1 File — (ZIP) [file pone.0219608.s002.zip › S1 Fig/No.18.tif]

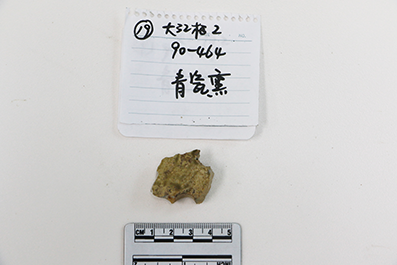

Supplement: S1 File — (ZIP) [file pone.0219608.s002.zip › S1 Fig/No.19.tif]

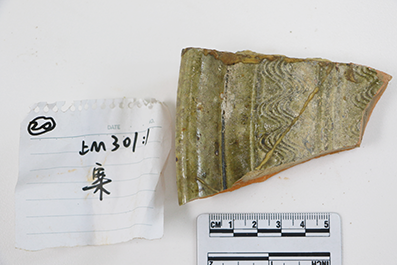

Supplement: S1 File — (ZIP) [file pone.0219608.s002.zip › S1 Fig/No.20.tif]

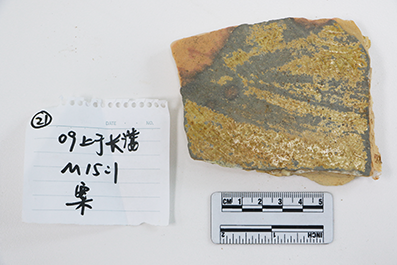

Supplement: S1 File — (ZIP) [file pone.0219608.s002.zip › S1 Fig/No.21.tif]

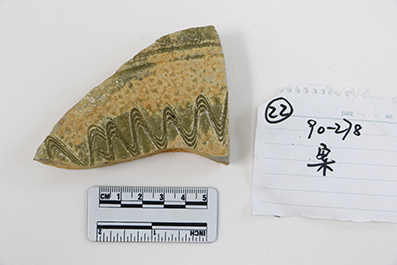

Supplement: S1 File — (ZIP) [file pone.0219608.s002.zip › S1 Fig/No.22.tif]

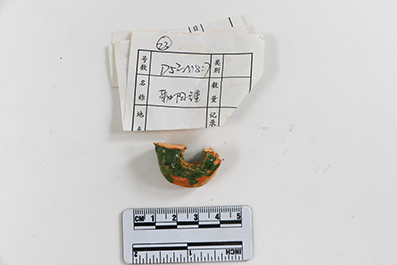

Supplement: S1 File — (ZIP) [file pone.0219608.s002.zip › S1 Fig/No.23.tif]

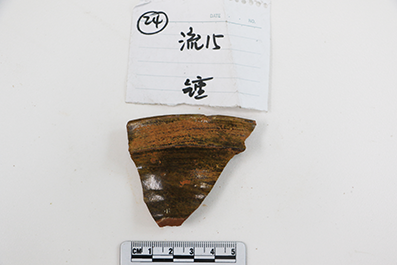

Supplement: S1 File — (ZIP) [file pone.0219608.s002.zip › S1 Fig/No.24.tif]

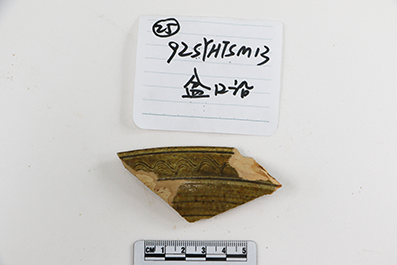

Supplement: S1 File — (ZIP) [file pone.0219608.s002.zip › S1 Fig/No.25.tif]

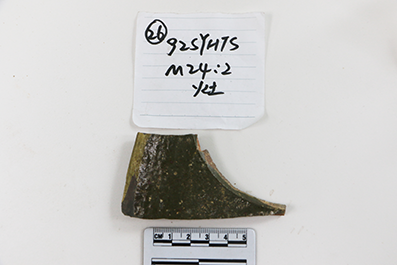

Supplement: S1 File — (ZIP) [file pone.0219608.s002.zip › S1 Fig/No.26.tif]

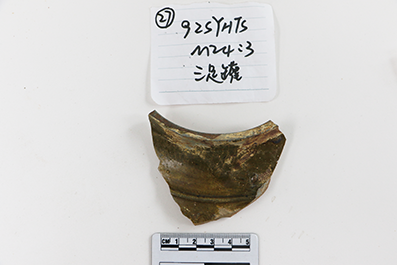

Supplement: S1 File — (ZIP) [file pone.0219608.s002.zip › S1 Fig/No.27.tif]

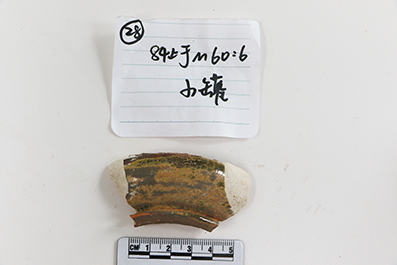

Supplement: S1 File — (ZIP) [file pone.0219608.s002.zip › S1 Fig/No.28.tif]

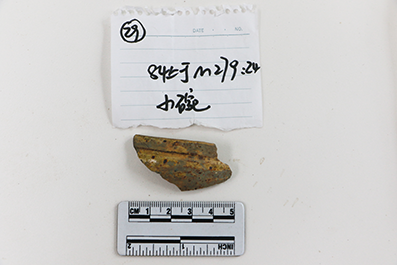

Supplement: S1 File — (ZIP) [file pone.0219608.s002.zip › S1 Fig/No.29.tif]

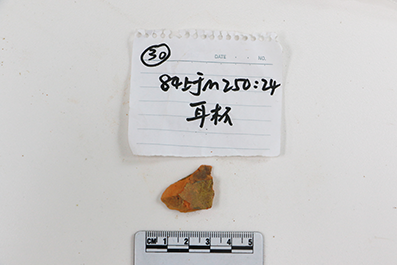

Supplement: S1 File — (ZIP) [file pone.0219608.s002.zip › S1 Fig/No.30.tif]

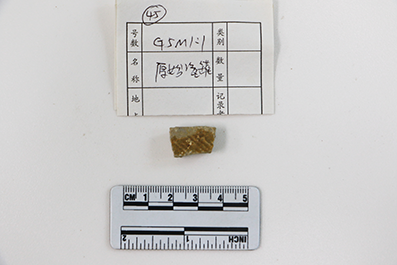

Supplement: S1 File — (ZIP) [file pone.0219608.s002.zip › S1 Fig/No.45.tif]

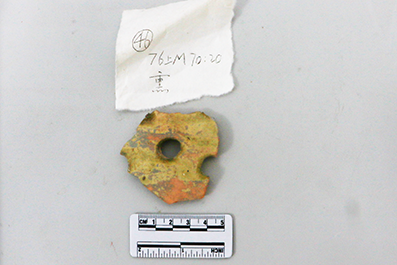

Supplement: S1 File — (ZIP) [file pone.0219608.s002.zip › S1 Fig/No.46.tif]

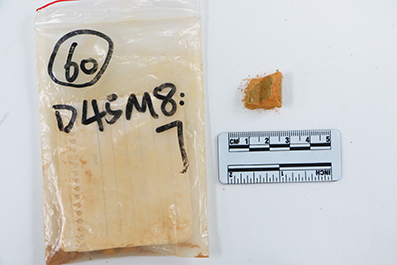

Supplement: S1 File — (ZIP) [file pone.0219608.s002.zip › S1 Fig/No.60.tif]

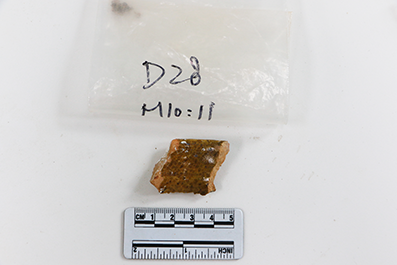

Supplement: S1 File — (ZIP) [file pone.0219608.s002.zip › S1 Fig/No.61.tif]
