# Supplementary material for: A technological combination of lead-glaze and calcium-glaze recently found in China: Scientific comparative analysis of glazed ceramics from Shangyu, Zhejiang Province
Source: PLoS One. 2019 Jul 11;14(7):e0219608. doi: 10.1371/journal.pone.0219608 (PMC6622541; doi:10.1371/journal.pone.0219608)
Supplement: S1 Table — (DOCX) [file pone.0219608.s003.docx]

**S1 Table. The LA-ICP-AES test results of the standard samples.**

|  | **Na_2_O** | **Mg _2_O** | **Al_2_O_3_** | **SiO_2_** | **P_2_O_5_** | **K_2_O** | **CaO** | **TiO_2_** | **Fe_2_O_3_** |
| --- | --- | --- | --- | --- | --- | --- | --- | --- | --- |
| **nist610** | 13.10 | 0.09 | 2.21 | 71.61 | 0.07 | - | 11.83 | 0.08 | 0.09 |
| **certified value** | 14 | 0.08 | 2 | 72 | 0.08 | 0.04 | 12 | 0.07 | 0.07 |
| **recovery%** | 94 | 113 | 110 | 99 | 90 |  | 99 | 118 | 122 |
|  |  |  |  |  |  |  |  |  |  |
|  |  |  |  |  |  |  |  |  |  |
| **corning-b** | 16.14 | 1.06 | 4.61 | 62.67 | 0.90 | 0.95 | 9.01 | 0.11 | 0.39 |
| **certified value** | 17 | 1.03 | 4.36 | 61.55 | 0.82 | 1 | 8.56 | 0.089 | 0.34 |
| **recovery%** | 95 | 103 | 106 | 102 | 110 | 95 | 105 | 124 | 114 |
|  |  |  |  |  |  |  |  |  |  |
| **sgt-II** | 13.01 | 2.21 | 1.90 | 71.13 | - | 0.55 | 10.34 | 0.07 | 0.38 |
| **certified value** | 13.6 | 2.14 | 1.83 | 70.7 |  | 0.69 | 10.3 | 0.068 | 0.342 |
| **recovery%** | 96 | 103 | 104 | 101 |  | 79 | 100 | 98 | 111 |
|  |  |  |  |  |  |  |  |  |  |
| **dgg-II** | 13.09 | 3.49 | - | 72.77 | - | - | 10.28 | 0.03 | 0.04 |
| **certified value** | 13.78 | 3.40 | 0.10 | 72.26 |  |  | 10.05 | 0.033 |  |
| **recovery%** | 95 | 103 |  | 101 |  |  | 102 | 98 | 0 |
